# Dynamic Temporal Change of Cerebral Microbleeds: Long-Term Follow-Up MRI Study

**DOI:** 10.1371/journal.pone.0025930

**Published:** 2011-10-11

**Authors:** Seung-Hoon Lee, Soon-Tae Lee, Beom Joon Kim, Hee-Kwon Park, Chi-Kyung Kim, Keun-Hwa Jung, Jae-Kyu Roh

**Affiliations:** 1 Department of Neurology, Seoul National University College of Medicine, Seoul, Republic of Korea; 2 Clinical Research Center for Stroke, Seoul National University Hospital, Seoul, Republic of Korea; 3 Department of Neurology, Inha University Hospital, Incheon, Republic of Korea; Lerner Research Institute, Cleveland Clinic, United States of America

## Abstract

**Background:**

Cerebral microbleeds (MBs) are understood as an important radiologic marker of intracerebral hemorrhage. We sought to investigate the temporal changes of MBs and clinical factors associated with the changes using long-term follow-up MRI.

**Methods/Principal Findings:**

From October 2002 to July 2006, we prospectively enrolled patients with stroke or transient ischemic attack, and followed-up their brain MRIs with an interval >12 mo. We compared demographic factors, vascular risk factors, laboratory findings, and radiologic factors according to the presence or changes of MBs. A total of 224 patients successfully completed the follow-up examinations (mean, 27 months). Newly developed MBs were noted in 10 patients (6.8%) among those without MBs at baseline (n = 148), and in those with MBs at baseline (n = 76), the MB count had decreased in 11 patients (14.5%), and increased in 41 patients (53.9%). The estimated annual rate of change of MB numbers was 0.80 lesions per year in all patients, a value which became greater in those patients who exhibited MBs at baseline (MBs≥5, 5.43 lesions per year). Strokes due to small vessel occlusion and intracerebral hemorrhage, as well as white matter lesions were independently associated with an increased MB count, whereas the highest quartile of low-density lipoprotein (LDL) cholesterol was associated with a decreased MB count.

**Conclusion:**

During the follow-up period, most of MBs showed dynamic temporal change. Symptomatic or asymptomatic small vessel diseases appear to act as risk factors while in contrast, a high level of LDL cholesterol may act as a protective factor against MB increase.

## Introduction

Cerebral microbleeds (MBs), which are seen as small, focal, dark signal intensity lesions on T2*-weighted gradient-echo magnetic resonance imaging (MRI), pathologically represent the perivascular extravasation of blood resulting from advanced cerebral microangiopathy, such as lipohyalinosis [1]. Old age [2], chronic hypertension [3], left ventricular hypertrophy [4], low serum cholesterol [5], and cerebral amyloid angiopathy [6] may be associated with the presence or increase of MBs. Further these lesions may be associated with the risks of future intracerebral hemorrhage with numerical and regional associations [7], and aspirin or warfarin-associated intracerebral hemorrhage [8], [9], thus serving a role as a risk factor or at least a radiological risk marker. In addition, as hemorrhage-type microangiopathy, MBs are positively correlated with the radiologic findings of ischemia-type microangiopathy - silent lacunar infarction and white matter lesions - in terms of the lesion extent; however, they are relatively different in terms of spatial distribution [10]. Furthermore, it has been suggested that MBs may play an active role in cognitive function [11]. Generally, there is increasing evidence relating to the importance of MB detection in various aspects of clinical practice.

Despite its suggested importance, most previous studies have been based on cross-sectional design which is questionable given that the longitudinal temporal changes of MBs have yet to be elucidated. In the current follow-up MRI study, we observed temporal changes of MBs in a prospective series from a stroke population, and attempted to determine factors associated with lesion changes.

## Methods

### Study design

In order to elucidate the long-term temporal changes of MBs after stroke or transient ischemic attack (TIA), we designed a long-term retrospective cohort study. From October 2002, we enrolled stroke or TIA patients who had been admitted to the stroke care unit of our hospital. We restricted the study population to patients who had been admitted within seven days of onset. At baseline, all patients underwent a complete set of clinical tests – past medical history, neurological examination, National Institutes of Health Stroke Scale (NIHSS) scoring, and basic laboratory tests for stroke. Further, all patients received standard stroke and best medical therapy during hospitalization. Follow-up brain MRIs were conducted between December 2007 and February 2008, with an interval of at least 12 months from the initial MRIs. If a patient had already undergone a brain MRI due to various medical conditions in the six months prior to their follow-up MRI visit, we did not perform the MRI again. Conduction of follow-up MRI with an interval of 12 months after stroke is covered by the National Health Insurance System in Korea, and we obtained informed consents from the participants verbally. We obtained medical information of the included patients from the electronic medical record system of our hospital. When lost to follow-up, we contacted the patient or the family members by telephone to confirm their status. Despite this effort, if a patient's status could not be confirmed, we classified the case as follow-up loss. In cases when the patient had expired, the date and cause of death according to the International Classification of Diseases (10th Revision), were recorded. To confirm the accuracy of mortality information, we matched our data to the nationwide official data on death certification provided by the National Statistical Office, data which have been used in previous studies as reliable data [12]. This study was approved by the Institutional Review Board of Seoul National University Hospital (H-0805-035-243).

### Determination of clinical variables and laboratory tests

We recorded demographic data (age and gender), conventional risk factors, and important laboratory data for all subjects. Conventional risk factors included hypertension, diabetes, hyperlipidemia, and being a current smoker. Hypertension was diagnosed as present if patients exhibited a systolic blood pressure >140 mm Hg or a diastolic blood pressure >90 mm Hg at discharge, or had a history of diagnosis of hypertension and anti-hypertensive medications. Diabetes was diagnosed as present if subjects exhibited a fasting glucose level ≥7.0 mmol/L (126 mg/dL) or had a history of diagnosis of diabetes and anti-diabetic medications. A diagnosis of hyperlipidemia was made in patients with a history of using cholesterol-lowering agents or who had a fasting serum total cholesterol level >6.2 mmol/L (240 mg/dL) at admission. Body mass index was calculated based on body weight and height (kg/m^2^). With the exception of hemorrhagic stroke, subtypes of ischemic stroke were determined by consensus from a panel of stroke neurologists the Trial of Org 10172 in Acute Stroke Treatment (TOAST) criteria with minor modifications [13]. Fasting blood samples were drawn within 24 hours of admission, and examined using a standard battery of biochemical and hematological tests. Lipid profiles included total cholesterol, low-density lipoprotein (LDL) cholesterol, high-density lipoprotein cholesterol, and triglycerides.

### Brain imaging protocols

Magnetic resonance images were obtained using a 1.5T superconducting magnet (GE Medical Systems, Milwaukee, WI, USA). In terms of protocol, the current study employed T2*-weighted gradient-echo [repetition time/echo time (TR/TE), 500/15 ms; flip angle, 26°; slice thickness, 5 mm; matrix size, 256×192) and fluid-attenuated inversion recovery (FLAIR; TR/TE, 8500/96 ms; inversion time, 2100 ms; flip angle, 26°; slice thickness, 5 mm; matrix size, 256×192) imaging.

In accordance with the recommended criteria [1], the MBs were defined as well-defined focal areas of dark signal intensity measuring less than 5 mm in diameter with blooming artifacts on gradient-echo MRI. We did not include lesions that were within the subarachnoid space or areas of symmetric hypointensity of the globus pallidus, given that they were likely to represent adjacent pial blood vessels and calcification, respectively. The numbers of the lesions were counted throughout the entire brain by two neurologists (BJ Kim and CK Kim) who were blind to the clinical characteristics.

White matter lesions were evaluated by FLAIR MRI, and classified into four grades: Grade 0, no abnormality or minimal periventricular signal hyperintensities in the form of caps confined exclusively to the anterior horns or rims lining the ventricles; Grade 1, hyperintensities in both the anterior and posterior horns of the lateral ventricles or periventricular unifocal patches; Grade 2, multiple periventricular hyperintense punctate lesions and their early confluence; and Grade 3, multiple areas of high signal intensity reaching confluence in the periventricular region [14].

### Statistical analysis

Means for continuous variables and proportions for categorical variables were compared using student *t*-tests and χ^2^-tests as appropriate. Data were expressed as means ± standard deviation (SD) or as numbers (percentages). To identify independent risk factors for MBs at baseline, univariate and multivariate logistic regression analysis was performed to calculate crude and adjusted odds ratios (ORs) with 95% confidence intervals (CIs). In this analysis, levels of lipid profiles were categorized into quartiles, and the highest or lowest values were used as appropriate, given that the relationships between values of lipid profiles and the presence of MBs were not linear. For comparisons among multiple groups from the patients with MBs at baseline (n = 76), a one-way analysis of variance (ANOVA) with Dunnett's post-hoc comparisons was used for continuous values, and the chi-square test was used for categorical variables.

To examine temporal change of MB, we calculated annual rates of change of MB counts by analyzing a mean of difference between numbers of MBs at follow-up and numbers at initial visit divided by the follow-up period (number of changes per year). In addition, we used generalized estimating equations (GEE) to evaluate the relationship between several predictor variables related to MB count [15]. These equations were necessary because of the clustered and correlated nature of counting MBs using two individual brain MRIs longitudinally over time. The repeated counting for each patient was treated as a cluster. Further, GEE models were estimated that used an exchangeable correlation matrix, assuming that outcomes for individual MR imaging were equally correlated within each patient but statistically independent between individuals. Univariable and multivariable analyses was performed using GEE models utilizing MB count as a dependent variable with Poisson log-linear link function. Clinically relevant variables or potentially significant variables from the univariate analyses (i.e. *p*<0.1) were subsequently chosen to construct a multivariable analysis model. Data produced by the GEE models were presented as rate ratios (RRs) with 95% CIs. All statistical analyses were conducted using SPSS for Windows, version 17.0 (SPSS Inc., Chicago, IL, USA), and statistical significance was accepted at the *p*<0.05 level.

## Results

### Study population

**Figure 1:**
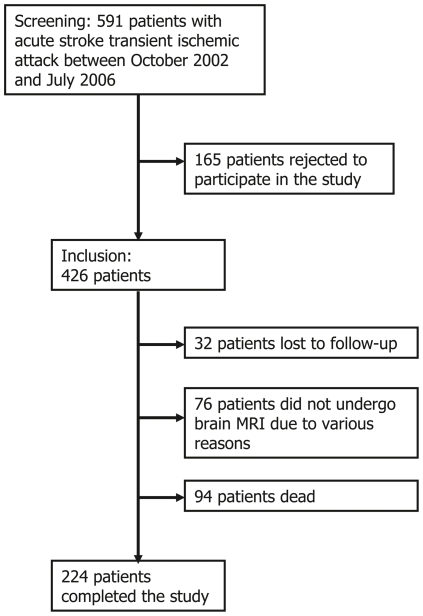
Flow chart for the study sample.

Initially, 591 patients with acute stroke or TIA were screened (Fig. 1). Among them, 165 patients refused participation in this study, and thus, 426 patients represented the final number of patients at baseline. Further, 32 patients (7.5%) were lost to follow-up, and 76 patients (17.8%) could not undergo follow-up brain MRI due to various medical reasons - irritability, claustrophobia, decreased performance, unstable medical condition, concomitant diseases, and patient delivery problem because of long distance between the resident area and our center. In addition, 94 patients (22.1%) expired during the study period. As a result, a total of 224 patients (52.6%) completed our study protocols and their data were included in the final analysis.

### Baseline characteristics

Among the 224 patients analyzed, there were 148 men (66.1%) and 76 women (33.9%), and ages ranged from 31 to 95 years (mean, 64.6±11.3 years). Intervals between initial and follow-up MRIs ranged from 12.0 to 62.6 months (mean, 27.5±12.3 months). At baseline, there were 209 patients with stroke and 22 patients with TIA. Stroke subtypes were as follows: 58 large-artery atherothromboses, 60 small-vessel occlusions, 33 cardioembolisms, 31 other determined or undetermined etiologies, and 20 intracerebral hemorrhages. On initial gradient-echo MRIs, 76 patients (33.9%) had cerebral MBs, and the numbers of MBs in the whole brain area ranged 0 to 81 (mean per patient, 3.4±10.5 lesions). When we compared the prevalence of MBs according to stroke subtypes, the highest was in intracerebral hemorrhage patients at 12 (57.1%). TIA represented the next highest prevalence with 4 (26.4% in all TIA patients), followed by large-artery atherothrombosis with 17 (28.8%), small-vessel occlusion with 23 (37.7%), cardioembolism with 9 (28.1%), and 8 with other determined or undetermined etiologies (26.7%). Baseline characteristics have been provided in Table 1. The patients with MBs at baseline showed a higher frequency of hypertension, lower frequency of diabetes, and lower levels of hemoglobin A1c than those patients without MBs.

**Table 1:** Baseline characteristics of patients according to presence of MBs at baseline.

	MBs at baseline	
	Absent (n = 148)	Present (n = 76)
Gender (men)	96 (64.9%)	52 (68.4%)
Age, years	64.8±11.2	64.5±11.5
Body mass index	23.8±3.1	23.5±3.1
Hypertension*	95 (64.2%)	59 (77.6%)
Diabetes*	60 (40.5%)	17 (22.4%)
Hyperlipidemia	27 (18.2%)	12 (15.8%)
Smoking	30 (20.3%)	16 (21.1%)
TIA	13 (8.8%)	9 (11.8%)
Stroke	135 (91.2%)	67 (88.2%)
NIHSS scores at baseline	2.1±2.1	2.8±5.0
Total cholesterol, mg/dL	183.9±46.4	175.3±39.7
LDL cholesterol, mg/dL	112.9±39.2	107.7±39.7
HDL cholesterol, mg/dL	44.8±12.6	41.7±11.8
Triglyceride, mg/dL	128.8±83.3	130.4±92.5
Hemoglobin A1c, %*	6.6±1.6	6.0±1.3
Creatinine, mg/dL	1.1±0.9	1.3±1.2
MBs, numbers	0	9.9±16.0

MB indicates microbleed; TIA, transient ischemic attack; NIHSS, National Institute of Health Stroke Scale; LDL, low-density lipoprotein; HDL, high-density lipoprotein; NS, not significant.

Student *t*-tests or χ^2^ tests were used.

*indicates *p*<0.05.

### Association factors for the presence of MBs at baseline

Using multivariable logistic regression analysis, we sought to determine factors associated with the presence of MBs at baseline. We entered demographic variables, vascular risk factors, some of initial laboratory findings (lipid profiles, hemoglobin A1c, and creatinine), and radiological findings (white matter lesions) after assuming that such laboratory findings and white matter lesions went unchanged after insult of stroke. The presence of diabetes was independently, but negatively associated with the presence of MBs (adjusted OR, 0.44; 95% CI, 0.22–0.90), whereas the severity of white matter lesions was a strong risk factor (adjusted OR, 2.32; 95% CI, 1.59–3.39).

### Long-term temporal changes of MBs

**Figure 2:**
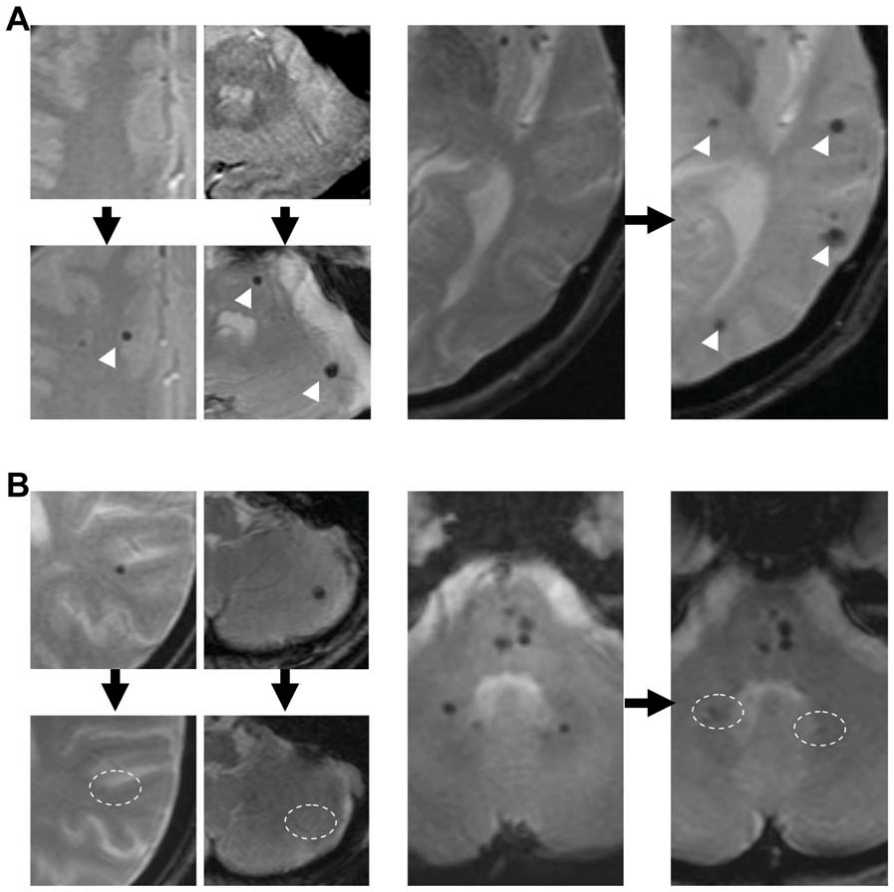
Dynamic changes of MBs over a long-term MRI follow-up. Some new MBs appeared (A), and some MBs disappeared in the follow-up MRI (B). Black arrows represent the MRI follow-up, while white arrow heads indicate new MBs, and dotted circles identify the location of those MBs which had disappeared.

Generally, there was an increasing trend of MBs during the study period. There was no patient, in which MBs at baseline completely disappeared at follow-up, whereas 10 patients (6.8%) among the patients without MBs at baseline (n = 148) had newly developed MBs at follow-up. Thus, a total of 86 patients had MBs at follow-up (38.4%), with the total number of MBs increasing by about 1.5-fold (mean, 4.9±16.8 lesions). Among the 76 patients with MBs at baseline, the numbers of MBs decreased in 11 (14.5%) patients, and increased in 41 (53.9%). The numbers of MBs in the remaining 24 (31.6%) patients were unchanged during the follow-up. Examples of dynamic temporal changes of MB have been provided in Fig. 2.

Table 2 and 3 provide the characteristics of these 76 patients with MBs at baseline. Using an ANOVA with post-hoc tests for intra- and inter-group comparisons, we found that those with decreased numbers of MBs exhibited higher total and LDL cholesterol levels at baseline (*p* = 0.030 and 0.018, respectively), and higher usage of angiotensin-converting enzyme inhibitors or angiotensin-receptor blockers (*p* = 0.027).

**Table 2:** Baseline characteristics of patients according to temporal change of MBs counts during follow-up: part 1.

		Number of MBs		
		Decrease (n = 11)	No change (n = 24)	Increase (n = 41)
Demographic	Gender (men)	6 (54.5%)	16 (66.7%)	30 (73.2%)
	Age, years	61.1±13.5	62.5±10.4	66.6±11.4
	Body mass index	23.7±3.5	24.2±2.5	22.9±3.3
Vascular risk factors	Hypertension	9 (81.8%)	19 (79.2%)	31 (75.6%)
	Diabetes	4 (36.4%)	5 (20.8%)	8 (19.5%)
	Hyperlipidemia	2 (18.2%)	5 (20.8%)	5 (12.2%)
	Smoking	4 (36.4%)	4 (16.7%)	8 (19.5%)
Clinical profiles	TIA	0 (0%)	4 (16.7%)	5 (12.2%)
	Stroke	11 (100%)	20 (83.3%)	36 (87.4%)
	NIHSS scores at baseline	2.2±2.9	2.4±1.9	3.1±6.4
Stroke subtypes	Small vessel occlusion	4 (36.4%)	7 (29.2%)	12 (29.3%)
	Large artery atherosclerosis	3 (27.3%)	7 (29.2%)	6 (14.6%)
	Cardioembolism	2 (18.2%)	2 (8.3%)	5 (12.2%)
	UD or OD	0 (0%)	1 (4.2%)	7 (17.1%)
	Intracerebral hemorrhage	2 (18.2%)	3 (12.5%)	6 (14.6%)

MB indicates microbleed; TIA, transient ischemic attack; NIHSS, National Institute of Health Stroke Scale; UD, undetermined; OD, other determined; ANOVA tests with Dunnett's post-hoc analysis or χ^2^ tests were used, but there were no significant results.

**Table 3:** Baseline characteristics of patients according to temporal change of MBs counts during follow-up: part 2.

		Number of MBs		
		Decrease (n = 11)	No change (n = 24)	Increase (n = 41)
Laboratory findings	Total cholesterol, mg/dL†	201.7±31.7	171.1±39.4	179.7±40.0
	LDL cholesterol, mg/dL* †	133.5±23.5	104.3±35.5	102.8±31.7
	HDL cholesterol, mg/dL	41.6±7.2	42.4±12.4	41.3±12.7
	Triglyceride, mg/dL	138.0±70.3	123.5±93.8	132.4±98.4
	Hemoglobin A1c, %	6.5±1.2	6.0±1.2	6.0±1.3
	Creatinine, mg/dL	1.1±0.3	1.1±0.5	1.5±1.5
Radiologic findings	White matter lesions, grade	2 (18.2%)	4 (16.7%)	7 (17.1%)
	Grade 0	2 (18.2%)	14 (58.3%)	13 (31.7%)
	Grade 1† §	4 (36.4%)	5 (20.8%)	10 (24.4%)
	Grade 2	3 (27.3%)	1 (4.2%)	11 (26.8%)
	Grade 3† §	2 (18.2%)	4 (16.7%)	7 (17.1%)
Medications	ACEIs/ARBs†	9 (81.8%)	10 (41.7%)	18 (43.9%)
	CCBs	4 (36.4%)	6 (25.0%)	7 (17.1%)
	Beta-blockers	2 (18.2%)	6 (25.0%)	10 (24.4%)
	Diuretics	7 (63.6%)	14 (58.3%)	10 (24.4%)
	Statins	4 (36.4%)	6 (25.0%)	12 (29.3%)
	Antiplatelet agents	6 (54.5%)	19 (79.2%)	29 (70.7%)
	Anticoagulation	2 (18.2%)	2 (8.3%)	8 (19.5%)
	Oral hypoglycemic agents	2 (18.2%)	3 (12.5%)	4 (9.8%)

LDL indicates low-density lipoprotein; HDL, high-density lipoprotein; ACEI, angiotensin-converting enzyme inhibitor; ARB, angiotensin-receptor blocker.

ANOVA tests with Dunnett's post-hoc analysis or χ^2^ tests were used:

*indicates *p*<0.05 from overall comparisons;

† , *p*<0.05 from post-hoc analysis between “decreased” and “no change”;

§ , *p*<0.05 from post-hoc analysis between “increased” and “no change”.

Estimated annual change rates of MB numbers are revealed in Table 4. The rate of increase in the number of MBs was about 0.8 lesions per year in all patients, a value which increased when we restricted our analysis only to those patients with MBs at baseline. The rate of increase in patients without MB at baseline was very small (0.04 lesions per year).

**Table 4:** Estimated annual rates of change of MB counts.

Patients	Annual rate of change of MB counts (lesions per year)
Total patients (n = 224)	0.80±3.80
MBs at baseline = 0 (n = 148)	0.04±0.14
MBs at baseline≥1 (n = 76)	2.30±6.28
MBs at baseline≥2 (n = 55)	3.00±7.25
MBs at baseline≥5 (n = 28)	5.43±9.59

MB indicates microbleed.

### Predictors of temporal changes of MBs

We used the GEE models to find predictors of temporal changes of MBs. Based on the univariable analyses, age and grades of white matter lesions were identified as predictors for increasing MB count, and diabetes, hyperlipidemia, the highest quartile of LDL-cholesterol, and the highest quartile of triglyceride during the follow-up period were documented as predictors for decreasing number of MBs (Table 5). Compared to stroke due to other types or transient ischemic attack, it was determined that small vessel occlusion and intracerebral hemorrhage were strongly associated with MB count. Based on the multivariable analyses performed to identify predictor variables for MB count over time, strokes due to small vessel occlusion and intracerebral hemorrhage, the highest quartile of LDL-cholesterol, and white matter lesions were determined to be independently associated with MB count (Table 6).

**Table 5:** Univariable analysis using the GEE model.

	Crude RR	95% CI
Gender (men)	1.21	0.53–2.77
Age (per 1-year increase)	1.04	1.01–1.07
Body mass index (per 1-kg/m^2^ increase)	0.97	0.85–1.12
Hypertension	1.67	0.52–5.29
Diabetes	0.32	0.13–0.76
Hyperlipidemia	0.35	0.13–0.97
Smoking	0.79	0.22–2.86
Small vessel occlusion	2.77	1.15–6.68
Intracerebral hemorrhage	4.72	1.61–13.84
NIHSS at admission (per 1-point increase)	0.99	0.89–1.10
Total cholesterol (4th quartile vs. others)	0.27	0.13–0.55
LDL-cholesterol (4th quartile vs. others)	0.23	0.11–0.49
HDL-cholesterol (1st quartile vs. others)	1.14	0.40–3.23
Triglyceride (4th quartile vs. others)	0.18	0.09–0.37
Hemoglobin A1c (per 1% increase)	0.79	0.67–0.94
Creatinine (per 1 mg/dL increase)	1.00	0.86–1.17
White matter lesions (per 1-grade increase)	2.78	1.62–4.78
ACEIs or ARBs	1.17	0.50–2.73
CCBs	0.71	0.32–1.56
Beta-blockers	1.77	0.68–4.60
Diuretics	1.30	0.44–3.83
Statins	0.90	0.32–2.56
Antiplatelet agents	0.81	0.33–2.01
Anticoagulation	0.60	0.23–1.57

GEE indicates generalized estimating equations; RR, rate ratio; CI, confidence interval; NIHSS, National Institute of Health Stroke Scale; LDL, low-density lipoprotein; HDL, high-density lipoprotein; ACEI, angiotensin-converting enzyme inhibitor; ARB, angiotensin-receptor blocker.

**Table 6:** Multivariable analysis using the GEE model.

	Adjusted RR	95% CI
Small vessel occlusion	2.49	1.15–5.40
Intracerebral hemorrhage	4.06	1.01–16.32
LDL-cholesterol (4^th^ quartile vs others)	0.30	0.14–0.68
White matter lesions (per 1-grade increase)	2.39	1.27–4.53

## Discussion

In this follow-up study, we found that temporal change in MB counts was dynamic with the total number of MBs increasing by 1.5-fold over time. The rate of change in MB counts was 0.80 lesions per year in all patients, and became much greater in patients with MBs at baseline. Specifically, this increasing trend was about 60-fold greater in patients with MBs at baseline than in patients without. Additionally, the presence of diabetes was negatively associated with the presence of MBs at baseline, whereas severity of white matter lesions was a strong risk factor. With regard to the change in MB counts during follow-up, strokes due to small vessel occlusion and intracerebral hemorrhage and white matter lesions were identified as independent risk factors, whereas the highest quartile of LDL cholesterol was identified as an independent protective factor.

MBs are closely associated with incident or recurrent intracerebral hemorrhage among the stroke subtypes, and it has been noted that the presence or number of MBs is the most important radiologic marker of a cerebral bleeding tendency due to small vessel disease [1], [16], [17]. However, long-term temporal change of lesions in the elderly has rarely been reported. A recent report examined 21 patients with ischemic stroke or TIA over an average of 5.5 years [18]. Their results showed that those with new MBs represented 50% of the patients with MBs at baseline (4 out of 8 patients); however, only 8% of patients without (1 out of 13 patients). In the present study, we showed that new MBs were found in 53.9% of patients with MBs at baseline (41 out of 76 patients), but only in 6.8% of patients without (10 out of 148 patients). Considering a follow-up period of 27 months, the current results regarding temporal change of MBs after ischemic stroke or TIA are in line with previous studies. Additionally, the great discrepancy regarding the increased incidence of MBs in patients with MBs at baseline as compared to those without MBs is very similar to the results from the PROGRESS MRI sub-study on temporal change of white matter lesions after ischemic stroke [19]. Ischemic stroke and TIA are frequently found in elderly people, and share substantial pathophysiological mechanisms although representing two different aspects of small vessel disease (ischemia vs. hemorrhage) [20], [21]. Results both from our study and the PROGRESS sub-study demonstrate that presence of small vessel disease at baseline itself may be crucial in progress of the small vessel disease [19]. Our results suggest that in patients with even a single MB, caution should be taken in their management, with special attention paid to the incidence of intracerebral hemorrhage.

We and others have previously shown that low concentrations of serum cholesterol are associated with the severe MBs, suggesting the antagonizing role of cholesterol in MB pathogenesis [5], [22]. In the present study, we found that the highest quartile of LDL cholesterol was independently associated with a decrease in MB counts during the follow-up. In fact, many large cohort studies have demonstrated that low serum levels of total cholesterol may represent a risk factor for intracerebral hemorrhage, especially in Japan [23], [24], [25], [26]. A similar inverse relationship was reported between serum total cholesterol level and the risk of death from hemorrhagic stroke in western countries [27]. Considering the pathology of MB (hemosiderin pigment accumulations in macrophages adjacent to the ruptured atherosclerotic microvessels) [21], the disappearance of MBs might depend on the clearance of hemosiderin-containing macrophages. Because MBs are frequently associated with microaneurysms [28], any changes related to hemosiderin-containing microaneurysms may be associated as well. It was hypothesized that high levels of LDL cholesterol were likely to prevent small vessel rupture in the brain [5], [29], [30]; however, there has been no evidence that high level of LDL cholesterol can enhance the clearance of hemosiderin-containing microglia. Taken together, these hypotheses warrant further confirmation studies.

Despite the accumulation of evidence on cholesterol and bleeding, association between the use of lipid-lowering therapy such as statin and the occurrence of cerebral bleeding episodes has been controversial. Only one report has indicated that intensive lipid-lowering after stroke significantly increased the incidence of intracerebral hemorrhage [31], whereas other statin studies have produced contradictory. While many issues exist (primary or secondary prevention of stroke, stroke subtypes, and risk factor burden, etc), systematic reviews on this topic show inconsistent conclusions [32], [33]. Therefore, the effects of low levels of LDL cholesterol on the brain should be further investigated in terms of the nature of the low level - intrinsic low level vs. low level therapeutically intervened by statin.

This study clearly demonstrated an association between small vessel occlusion and intracerebral hemorrhage as baseline stroke subtypes with an increase in MB counts. This result confirms that MBs are produced by advanced cerebral small vessel disease [10], [21]. Moreover, an association with small vessel occlusion, that is lacunar infarction, indicates caution should be taken with intensive long-term anti-thrombotic therapy in such patients.

There are some caveats to this study. First, in general, diabetes is not a risk factor for intracerebral hemorrhage [34]. In this study, the presence of diabetes was a protective factor for presence of MBs at baseline, but this association was not replicated in the follow-up analysis. It has been reported that diabetes tends to increase large artery atherosclerosis rather than small vessel occlusion [35], [36]; however, this suggestion was not supported here. The biological effects of diabetes on the brain vascular system need to be clarified. Second, from the initial population, 90 patients expired and thus, a suitable follow-up was not possible. It is possible that the deceased patients may have demonstrated a greater change in MB count than those patients that lived through the follow-up period. If we had performed frequent brain MRIs, we would have shown more a precise temporal course; however, this study design did not allow for that due to ethical and economical considerations. Thus, our study results may be interpreted for surviving patients during the follow up period after ischemic stroke and TIA.

Our results showed that long-term temporal change of MBs after ischemic stroke is dynamic, and that this dynamicity is largely affected by the initial appearance of MBs. Further, changes in MB count may be associated with small vessel disease and the level of LDL cholesterol. Our results provide an effective basis for further MB studies. Recent recognition of the effect of MB on cognitive decline indicates that MBs are no longer a “silent” lesion. Thus, dynamic changes in MB counts should be further investigated in terms of their effects on cognitive function as well as on other vascular events.
